# Long-Lasting Graft-Derived Donor T Cells Contribute to the Pathogenesis of Chronic Graft-versus-Host Disease in Mice

**DOI:** 10.3389/fimmu.2017.01842

**Published:** 2017-12-18

**Authors:** Mizuha Kosugi-Kanaya, Satoshi Ueha, Jun Abe, Shigeyuki Shichino, Francis H. W. Shand, Teppei Morikawa, Makoto Kurachi, Yusuke Shono, Naoto Sudo, Ai Yamashita, Fumiko Suenaga, Akihiro Yokoyama, Wang Yong, Masahiro Imamura, Takanori Teshima, Kouji Matsushima

**Affiliations:** ^1^Department of Molecular Preventive Medicine, Graduate School of Medicine, The University of Tokyo, Tokyo, Japan; ^2^CREST, Japan Science and Technology Agency, Tokyo, Japan; ^3^Department of Hematology, Hokkaido University Graduate School of Medicine, Sapporo, Japan; ^4^Department of Pathology, Graduate School of Medicine, The University of Tokyo, Tokyo, Japan; ^5^Department of Microbiology, University of Pennsylvania School of Medicine, Philadelphia, PA, United States; ^6^Department of Immunology, Memorial Sloan-Kettering Cancer Center, New York, NY, United States; ^7^Department of Hematology, Sapporo Hokuyu Hospital, Sapporo, Japan

**Keywords:** chronic graft-versus-host disease, stem cell transplantation, T cell, T cell subset, immune reconstitution

## Abstract

Chronic graft-versus-host disease (cGVHD) is a major complication in long-term survivors of allogeneic hematopoietic stem cell transplantation (allo-HSCT). Graft-derived T cells (T_G_) have been implicated in the induction of cGVHD; however, the extent of their contribution to the pathogenesis of cGVHD remains unclear. Using a mouse model of cGVHD, we demonstrate that T_G_ predominate over hematopoietic stem cell-derived T cells generated *de novo* (T_HSC_) in cGVHD-affected organs such as the liver and lung even at day 63 after allo-HSCT. Persisting T_G_, in particular CD8^+^ T_G_, not only displayed an exhausted or senescent phenotype but also contained a substantial proportion of cells that had the potential to proliferate and produce inflammatory cytokines. Host antigens indirectly presented by donor HSC-derived hematopoietic cells were involved in the maintenance of T_G_ in the reconstituted host. Selective depletion of T_G_ in the chronic phase of disease resulted in the expansion of T_HSC_ and thus neither the survival nor histopathology of cGVHD was ameliorated. On the other hand, T_HSC_ depletion caused activation of T_G_ and resulted in a lethal T_G_-mediated exacerbation of GVHD. The findings presented here clarify the pathological role of long-lasting T_G_ in cGVHD.

## Introduction

Chronic graft-versus-host disease (cGVHD) remains a major cause of long-term morbidity, mortality, and poor quality of life following allogeneic hematopoietic stem cell transplantation (allo-HSCT) (1, 2). One hurdle in the development of preventive and therapeutic strategies against cGVHD is the complex origins and maintenance mechanisms of the pathogenic immune cells that are involved in this protracted disease. Despite clinical and experimental evidence that mature T cells in the graft (T_G_) are responsible for tissue injury during acute GVHD (aGVHD), the contribution of T_G_ to the pathogenesis of cGVHD remains poorly understood.

After allo-HSCT, host-reactive T_G_ are primed by host-type professional antigen-presenting cells (APCs) *via* the direct allo-recognition pathway in the secondary lymphoid organs, where they expand and differentiate into host-reactive effector T_G_ (3, 4). Effector T_G_ then redistribute to the target organs of aGVHD, such as the skin, liver, intestine, and hematopoietic and lymphoid tissues, where they impair host tissue function. Of note, an indirect allo-recognition pathway in which donor bone marrow (BM)-derived APCs repopulate peripheral tissues, uptake host-type antigens, and present them to donor T cells maximizes GVHD in a CD8-dependent GVHD model (5). Interestingly, *in vivo* early T cell depletion by antithymocyte globulin (6, 7) or post-transplant cyclophosphamide (8–10) reduces cGVHD rather than aGVHD. These clinical observations suggest a role for T_G_ in the development of cGVHD, but this is difficult to examine in a human setting, and experimental evidence demonstrating the contribution of long-lasting T_G_ to the development of cGVHD is lacking.

Since non-hematopoietic cells in the target organs remain as host-type even after allo-HSCT, T_G_ are continuously exposed to cognate antigens, which theoretically induce deletion, anergy, or replicative senescence in T_G_ (11, 12). In contrast to T_G_, which lack *de novo* replenishment, donor HSC-derived T cells (T_HSC_) that have undergone thymic selection are continuously replenished from the thymus, and thus T_HSC_ rather than T_G_ have been implicated in the pathogenesis of cGVHD (13–15). On the other hand, Hossain et al. (16) have shown that functional T_G_ persisted up to 100 days after allo-HSCT in a cGVHD model and that the persisting T_G_ confer protection against murine cytomegalovirus infection. This finding suggests that persisting T_G_ could be a functional population with a role in the pathogenesis of cGVHD.

In the present study, using a minor-mismatched allo-HSCT model in which the GVHD recipients display histopathology characteristic of cGVHD, we characterized the kinetics, function, and antigen reactivity of T_G_ and T_HSC_. Selective depletion of T_G_ or T_HSC_ in the chronic phase of disease revealed that persisting T_G_ suppress the accumulation of T_HSC_ in cGVHD-affected organs, whereas T_HSC_ suppress the lethal activation of T_G_ in affected organs.

## Materials and Methods

### Mice

Female C57BL/6J (B6; H-2^b^, CD45.2, Thy1.2) mice were purchased from CLEA Japan. C57BL/6J.SJL (B6.SJL; Ptprca Pepcb, CD45.1, Thy1.2), C3H.SW-H2b (B6; H-2^b^, CD45.2, Thy1.2), and C57BL/6J-Igh^a^-Thy1^a^-Gpi^a^ (B6.Thy1a; CD45.2, Thy1.1) mice were purchased from the Jackson Laboratory. B6 background congenic strains were crossed in-house to obtain CD45.1^+^ CD45.2^+^ Thy1.2^+^ and CD45.1^+^ CD45.2^+^ Thy1.1^+^ congenic strains. All animal experiments were conducted in accordance with institutional guidelines with the approval of the Animal Care and Use Committee of the University of Tokyo.

### Transplantation and Assessment of GVHD

Cell preparation and allo-HSCT were performed as described previously (17, 18) with some modifications. In brief, T-cell-depleted BM (TCD BM) was prepared by depleting Thy1^+^ mature T cells from BM using an autoMACS system (Miltenyi Biotec). Splenic T cells were negatively enriched from splenocytes by autoMACS, using antibodies against CD11b, CD11c, B220, Ter-119, NK1.1, and c-kit. Recipients were lethally irradiated (9 Gy, split into two doses given 3 h apart) on day −1, then injected intravenously with 5 × 10^6^ TCD BM cells with or without 3–4 × 10^6^ splenic T cells on day 0. The development of systemic GVHD was quantified by measuring weight loss and using a clinical GVHD scoring system, as described previously (19).

### Histological Analyses

For the assessment of pathological changes in tissues, 4–6-µm formalin-fixed paraffin sections were stained with H&E and assessed by a pathologist (Teppei Morikawa; blinded to experimental group) using a scoring system described previously (20–22). For immunohistological analyses, the left lobes of the lung were inflated by infusion of 500 µl OCT compound intratracheally before lung tissue was harvested. Acetone-fixed 6- to 8-µm cryosections were incubated sequentially with primary antibodies and the appropriate fluorochrome-labeled secondary antibodies after blocking. Sections were mounted with Prolong Gold Antifade Reagent (Life Technologies) and visualized using an SP-5 confocal microscope (Leica Microsystems).

### Cell Preparation

Single cell suspensions were prepared from the liver, lung, spleen, BM and thymus after systemic transcardial perfusion with PBS. Liver cells were prepared by pressing liver tissue through a 200-µm stainless steel mesh (23). The right lobe of lung tissue was cut into small fragments and digested for 1 h at 37°C with 0.2% collagenase D (Roche, Penzberg, Germany) and 2,000 U/ml DNase I (Calbiochem, La Jolla, CA, USA). BM cells were flushed from femurs using a needle and syringe. Spleen and thymus tissue were pressed through a 70-µm cell strainer. Non-hematopoietic cells and cell debris were removed by 40% Percoll (GE healthcare) phase separation, and erythrocytes were removed using ACK lysing buffer.

### Flow Cytometry

Labeled and purified antibodies were purchased from BD Biosciences, BioLegend, or eBioscience (Table 1). Single cell suspensions were incubated sequentially with anti-CD16/32 (to block Fc receptors) then primary antibodies. Data were collected on a Gallios flow cytometer (Beckman Coulter) and analyzed using FlowJo software (Tree Star). For intracellular cytokine analysis, 5–10 × 10^5^ leukocytes were restimulated with ionomycin (1 µg/ml) and PMA (25 ng/ml) in the presence of Brefeldin A (10 µg/ml) for 4.5–5 h at 37°C. Following staining for surface antigens, cells were stained for intracellular cytokines using a Cytofix/Cytoperm kit (BD Bioscience), according to the manufacturer’s instructions. For short-term pulse BrdU labeling, mice were injected intraperitoneally with 1 mg/mouse BrdU (Sigma-Aldrich) in 100 µl PBS 1 h before sacrifice. BrdU incorporation was examined using a BrdU flow kit (BD Biosciences), according to the manufacturer’s instructions. For the intracellular staining of Foxp3 and Ki-67, 1 × 10^6^ leukocytes were stained for surface antigens and then were fixed and permeabilized with Foxp3 Fix/Perm Buffer (eBioscience) according to the manufacturer’s instructions.

**Table 1:** List of antibodies used for flow cytometry.

Antigen	Clone	Manufacturer
CD3ε	145-2C11	BD Biosciences
CD4	RM4-4	BioLegend
CD8α	53-6.7	BioLegend
CD11b	M1/70	BD Biosciences
CD16/32	2.4G2	BioXcell
CD25	PC61	BD Biosciences
CD44	IM7	BD Biosciences
CD45.1	A20	BioLegend
CD45.2	104	BD Biosciences
B220	RA3-6B2	BioLegend
CD48	HM48-1	BioLegend
CD62L	MEL-14	BD Biosciences
CD69	H1.2F3	BD Biosciences
CD90.1	OX-7	BioLegend
CD90.2	53-2.1	BD Biosciences
CD117 (c-Kit)	2B8	BD Biosciences
CD127 (IL-7Rα)	A7R34	BioLegend
CD150	TC15-12F12.2	BioLegend
Ly-6A/E (Sca-1)	D7	BioLegend
LAG-3	C9B7W	eBioscience
CD279 (PD-1)	J43	eBioscience
CD279 (PD-1)	RMP1-30	BioLegend
Ly-6C/G (Gr-1)	RB6-8C5	BioLegend
NK1.1	PK136	BD Biosciences
Ter-119	TER-119	BioLegend
Foxp3	FJK-16s	eBioscience
KLRG-1	2F1/KLRG1	BioLegend
BrdU	B44	BD Biosciences
Ki-67	SolA1s	eBioscience
TCR-β	H57-597	BD Biosciences
IFN-γ	XMG1.2	BioLegend
TNF-α	MP6-XT22	BD Biosciences
Granzyme B	GB11	BioLegend
Streptavidin		BioLegend
Collagen I	Rabbit anti-serum	LSL
Lyve-1	Goat IgG, polyclonal	R&D
PanEndothelial	MECA-32	BioLegend
Rabbit IgG	Donkey IgG, polyclonal	Thermo Fisher
Goat IgG	Donkey IgG, polyclonal	Thermo Fisher
Rat IgG	Donkey IgG, polyclonal	Thermo Fisher

### *Ex Vivo* and *In Vivo* Proliferation Assays

Chronic graft-versus-host disease was induced by transferring CD45.1^+^ Thy1.1^+^ TCD BM and CD45.1^+^ Thy1.2^+^ splenic T cells. Single cell suspensions of the liver and lung were prepared on day 49, and total T cells were enriched by negative selection with antibodies against CD11b, CD31, CD140a, CD326, B220, NK1.1, and Ter-119. T_G_ and T_HSC_ were enriched from the T cell fraction using positive selection with antibodies against Thy1.2 and Thy1.1, respectively, and used as secondary donors in *in vivo* proliferation assays, or as responders in the *ex vivo* proliferation assay. Non-hematopoietic cells were prepared from the lung of untreated C3H.SW-H2b mice or day 49 BMT recipients by negative selection with antibodies against CD45 and Ter-119. CD3^−^ NK1.1^−^ hematopoietic cells were prepared from the spleen of untreated C3H.SW-H2b mice or day 49 BMT recipients by negative selection with antibodies against CD3 and NK1.1. In an *ex vivo* proliferation assay, T_G_ and T_HSC_ were labeled with 5 µM CFSE and were cocultured with the non-hematopoietic cells or CD3^−^ NK1.1^−^ hematopoietic cells at a ratio of 1:10 for 3 days. In an *in vivo* proliferation assays, CFSE-labeled T_G_ and T_HSC_ were equally mixed and transferred intravenously into day 49 BMT recipients reconstituted with CD45.2 TCD BM.

### *In Vivo* T Cell Depletion

To deplete T_G_ or T_HSC_ selectively, recipient mice expressing various combinations of the congenic markers Thy1.1 and Thy1.2 were injected intraperitoneally with purified rat IgG2b anti-mouse Thy1.2 mAb (clone 30H12, BioXcell) on days 21 (200 µg) and 24 (100 µg) after allo-HSCT, followed by weekly administration (200 µg) from days 31–63.

### Statistical Analyses

All values are expressed as mean ± SEM. All data are representative of results obtained in at least two independent internally controlled experiments, where intergroup effects observed within each experiment were consistent across all experiments. Survival curves for GVHD were plotted using Kaplan–Meier estimates and compared by log-rank analysis (Prism version 5.0; GraphPad Prism Software). Comparisons between two groups were performed by unpaired two-tail Student’s *t*-tests. Multiple comparisons were performed by one-way ANOVA with Dunnett’s post-test. *P*-Values less than 0.05 were considered to be statistically significant.

## Results

### Establishment of a Clinically Relevant Model of cGVHD

**Figure 1:**
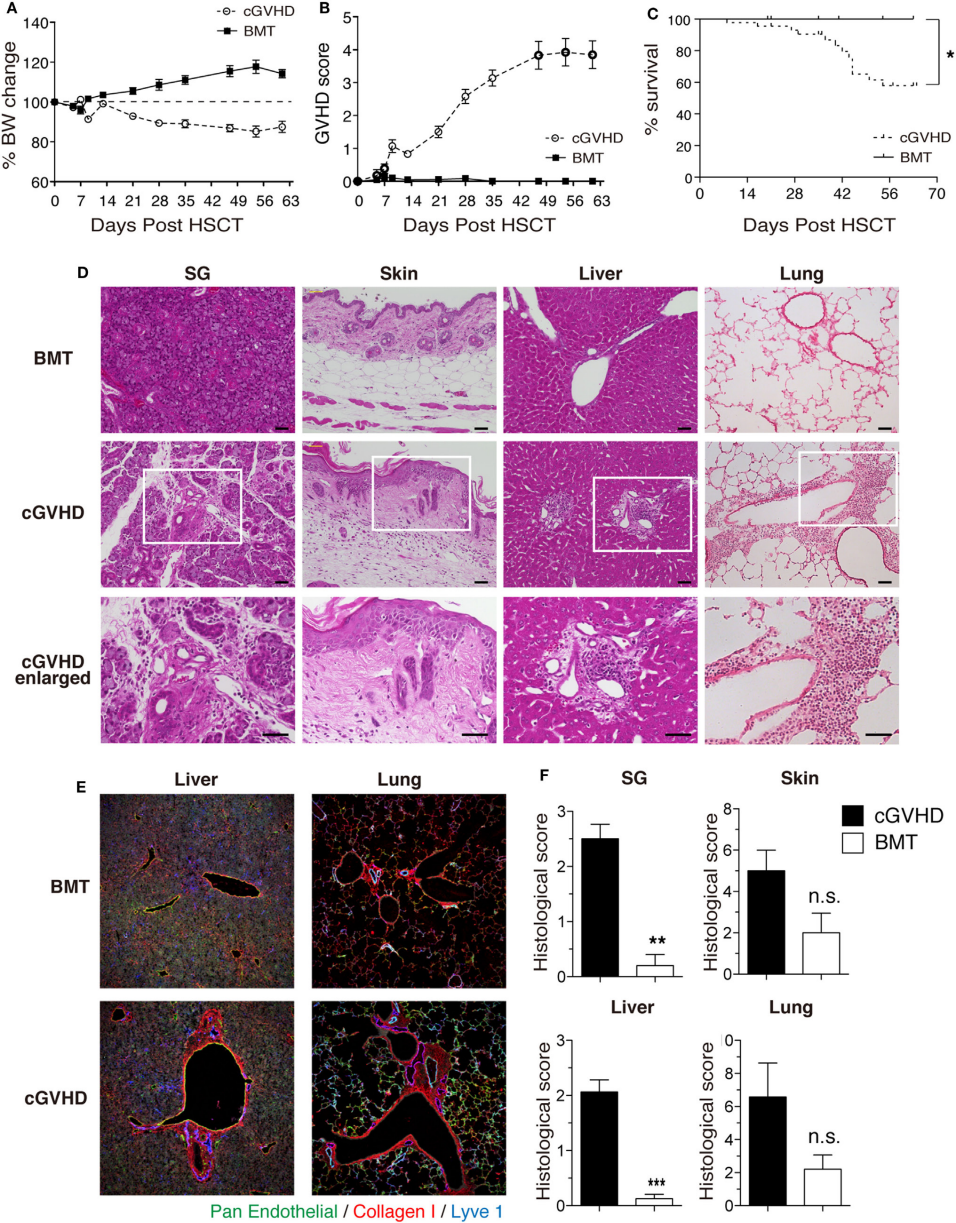
Establishment of a clinically relevant model of chronic graft-versus-host disease (cGVHD). Lethally irradiated C3H.SW mice were transplanted with T-cell-depleted bone marrow (TCD BM) alone or TCD BM plus 3–4 × 10^6^ T cells from the spleens of B6 mice. **(A)** Body weight, **(B)** GVHD score, and **(C)** survival were monitored for 63 days after transplantation. Data are combined from three independent experiments (*n* = 21 for BMT control group; *n* = 29 **(A,B)**; or *n* = 44 **(C)** for cGVHD group). **(D)** Salivary glands (SG), skin, liver, and lung were collected 63 days after transplantation for examination of pathology by HE staining. Scale bars, 50 µm. **(E)** Immunofluorescent staining of liver and lung sections. Green, pan-endothelial; red, collagen; and blue, Lyve1. **(F)** Histological scores from SG, skin, liver, and lung. Data are combined from two independent experiments (*n* = 5 for BMT control group; *n* = 8 for cGVHD groups). *P ≤ 0.05; **P ≤ 0.01; and ***P ≤ 0.001 (cGVHD vs. BMT).

We first established a B6→C3H.SW minor-mismatched cGVHD model to facilitate identification and manipulation of T_G_ and T_HSC_ populations using CD45 and Thy1 congenic markers. Lethally irradiated C3H.SW recipients were transplanted with TCD BM alone (“BMT group”) or TCD BM with splenic T cells (“cGVHD group”) from B6 donors. In the cGVHD group, body weight loss and GVHD score became progressively worse from day 14 onward (Figures 1A,B), and cGVHD mice had a significantly lower survival rate (Figure 1C). At day 63, the histology of the salivary glands (SG), skin, liver, and lung revealed inflammatory cell infiltration and fibrotic changes, which are the most relevant histological features in the diagnosis of human cGVHD (Figures 1D,E) (24, 25). The pathological scores for these organs were higher in cGVHD mice compared to BMT control mice, reaching statistical significance in the SG and liver (Figure 1F). These results demonstrate that cGVHD mice in the B6→C3H.SW minor-mismatched allo-HSCT model develop pathology that recapitulates human cGVHD.

### T_G_ Predominate over T_HSC_ in cGVHD-Affected Organs

**Figure 2:**
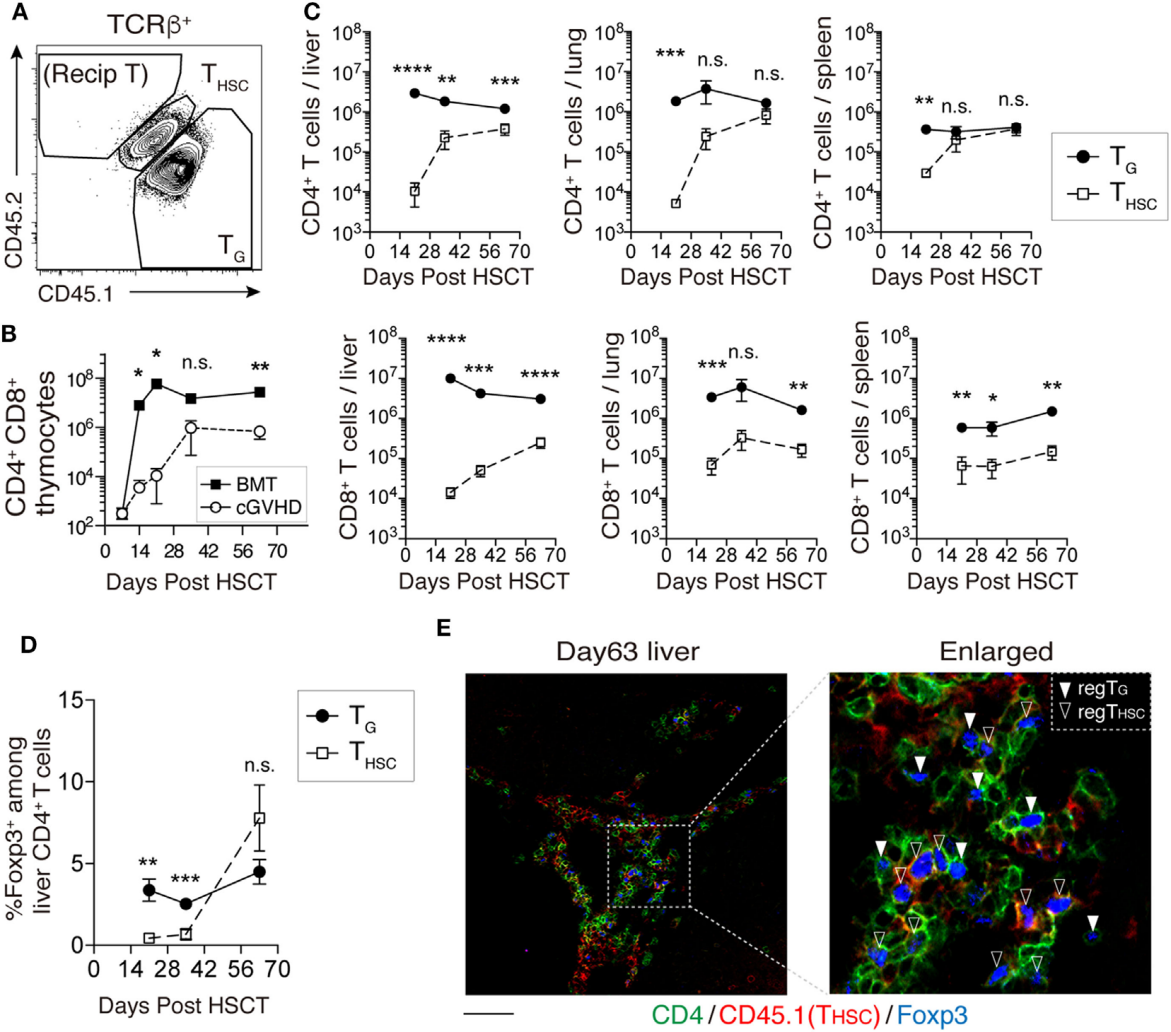
T_G_ predominate over T_HSC_ in chronic graft-versus-host disease (cGVHD)-affected organs. CD45.1^+^ CD45.2^+^ T-cell-depleted bone marrow (TCD BM) and CD45.1^+^ CD45.2^−^ spleen T cells from B6 donor mice were transferred into CD45.1^−^ CD45.2^+^ C3H.SW-recipient mice. **(A)** Representative flow cytometry plot of TCRβ^+^ T cells showing how congenic markers were used to detect T cells from distinct origins. CD45.1^+^ CD45.2^+^ cells represent T_HSC_ cells derived from BM HSC. CD45.1^+^ CD45.2^−^ T cells represent T_G_ derived from mature T cells in the graft. **(B)** The number of CD4^+^ CD8^+^ thymocytes of CD45.1^+^ CD45.2^+^ TCD BM origin (T_HSC_) was used as an indicator of immune reconstitution in the thymus. Representative data are shown from one of two independent experiments (*n* = 4). **(C)** Numbers of T_G_ and T_HSC_ collected from the liver (per gram), lung, and spleen following allogeneic hematopoietic stem cell transplantation (allo-HSCT). **(D)** The proportion of Foxp3^+^ cells within the liver CD4^+^ cell population following allo-HSCT. Data represent mean ± SEM (*n* = 5–7 per time point) and are combined from two independent experiments. **(E)** CD45.1^+^ TCD BM and CD45.2^+^ spleen T cells from B6 donor mice were transferred into C3H.SW-recipient mice, and the localization of CD4^+^ Foxp3^+^ T cells of CD45.1^+^ (T_HSC_) or CD45.1^−^ (T_G_) origin in the liver was analyzed on day 63. Green, CD4; red, CD45.1; and blue, Foxp3. Scale bar, 50 µm. *P ≤ 0.05; **P ≤ 0.01; ***P ≤ 0.001; and ****P ≤ 0.0001 [cGVHD vs. BMT **(B)** or T_G_ vs. T_HSC_ **(C)**].

We next examined the kinetics of T_G_ and T_HSC_ in our cGVHD model using CD45 congenic markers (Figure 2A). The number of CD4^+^ CD8^+^ thymocytes of CD45.1^+^ CD45.2^+^ TCD BM origin (T_HSC_) in cGVHD mice increased markedly between days 21 and 35 (Figure 2B). In peripheral tissues such as the liver, lung, and spleen, detectable numbers of T_HSC_ appeared from day 21 after HSCT, but within the CD8^+^ T cell populations of these tissues, T_G_ outnumbered T_HSC_ approximately 10-fold for the duration of our experiments (Figure 2C). A similar trend was observed in CD4^+^ T cell populations. Interestingly, T_G_ were more abundant than T_HSC_ within CD4^+^ Foxp3^+^ regulatory T cells in the liver by day 35; however, there was a trend toward T_HSC_ dominance by day 63 (Figure 2D). Immunofluorescent staining of the liver showed that both Foxp3^+^ CD4^+^ T_G_ and Foxp3^+^ CD4^+^ T_HSC_ were uniformly distributed throughout the T cell pool at day 63 (Figure 2E). These results suggest that T_G_ rather than T_HSC_ persist as the dominant CD8^+^ T cell population, while T_HSC_ gradually become the dominant CD4^+^ T cell population, particularly within the Foxp3^+^ CD4^+^ regulatory T cell populations in cGVHD-affected organs.

### T_G_ Remain Functional during cGVHD

**Figure 3:**
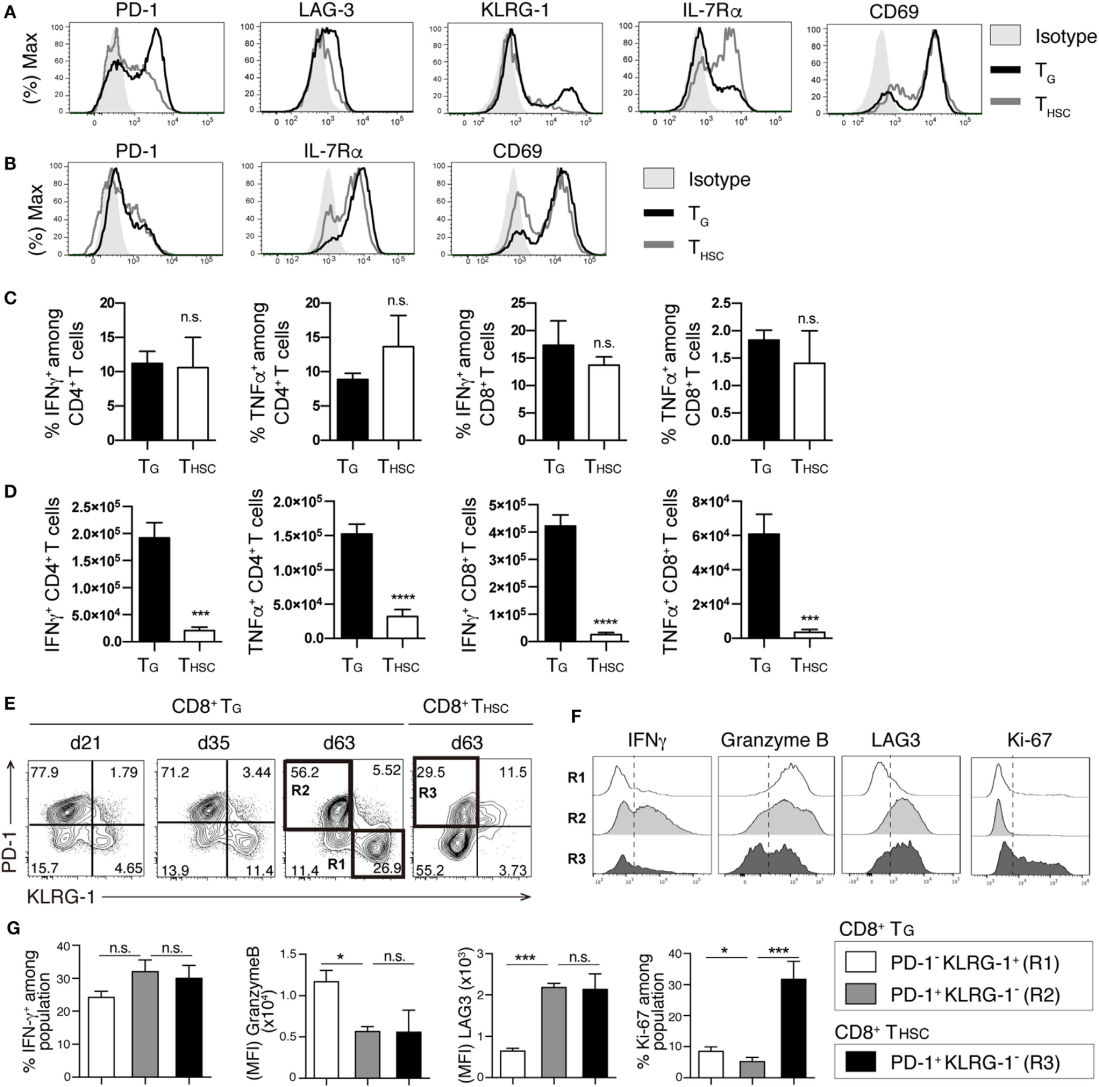
T_G_ remain functional during chronic graft-versus-host disease (cGVHD). T cells were collected from the liver 63 days after allogeneic hematopoietic stem cell transplantation (allo-HSCT). Immunophenotype of CD8^+^ **(A)** or CD4^+^ **(B)** T_G_ and T_HSC_ from cGVHD recipients. Representative data are shown from one of two independent experiments (*n* = 6). **(C,D)** Liver T cells were restimulated with PMA and ionomycin *ex vivo* and were analyzed for the intracellular IFNγ and TNFα 63 days after allo-HSCT. **(C)** The percentage of IFNγ^+^ or TNFα^+^ cells within the T_G_ or T_HSC_ populations and **(D)** the absolute numbers of these cells. **(E)** Time-course analyses of PD-1 and KLRG-1 expression on liver-infiltrating T_G_ and T_HSC_. **(F,G)** Populations R1, R2, and R3 from **(E)** were analyzed for the expression of IFNγ, Granzyme B, LAG-3, and Ki-67. Representative plots are shown from one of two independent experiments (*n* = 6). Data represent mean ± SEM (*n* = 6) from one of two independent experiments. *P ≤ 0.05; ***P ≤ 0.001; and ****P ≤ 0.0001.

We next compared the phenotype and function of T_G_ and T_HSC_ in the liver at day 63. Compared to their T_HSC_ counterparts, CD8^+^ T_G_ expressed high levels of the exhaustion markers PD-1 and LAG-3, the senescence/terminal differentiation marker KLRG-1 (11, 26), and low levels of IL-7Rα, which is inversely correlated with alloreactivity (27) (Figure 3A). Expression levels of the early activation marker CD69 were comparable between T_G_ and T_HSC_ (Figure 3A). However, we did not observe significant differences in surface marker expression between CD4^+^ T_G_ and CD4^+^ T_HSC_ (Figure 3B). Upon *ex vivo* stimulation of liver-infiltrating T cells with PMA and ionomycin, a substantial proportion of CD8^+^ T_G_ produced IFNγ and TNFα at levels that were equal to those produced by their T_HSC_ counterparts (Figure 3C). Because T_G_ far outnumbered T_HSC_ in cGVHD-affected organs, the majority of cells with potential to produce inflammatory cytokines in cGVHD-affected organs were of T_G_ origin (Figure 3D). A similar trend was observed for CD4^+^ T_G_ and T_HSC_ (Figures 3C,D). These results suggest that a substantial proportion of CD4^+^ and CD8^+^ T_G_ persist as a functional population in the target organs of cGVHD.

Interestingly, the proportion of PD-1^−^ KLRG-1^+^ cells within the CD8^+^ T_G_ population increased between day 21 and day 63 (Figure 3E). To examine whether these PD-1^−^ KLRG-1^+^ cells are functionally distinct from the PD-1^+^ KLRG-1^−^ cells that make up the majority of CD8^+^ T_G_ in early phase, we analyzed the expression of functional molecules and the proliferation marker Ki-67 by these two populations at day 60. The PD-1^+^ KLRG-1^−^ population displayed higher IFNγ production and LAG3 expression than the PD-1^−^ KLRG-1^+^ population, whereas the PD-1^−^ KLRG-1^+^ population displayed higher granzyme B and Ki-67 expression than the PD-1^+^ KLRG-1^−^ population (Figures 3F,G). We also compared the functional profiles of the PD-1^+^ KLRG-1^−^ populations from CD8^+^ T_G_ and T_HSC_. Although expression levels of LAG3 and granzyme B were equivalent between T_G_ and T_HSC_, PD-1^+^ KLRG-1^−^ T_HSC_ had lower IFNγ production and significantly higher expression of Ki-67 than their T_G_ counterparts (Figure 3G). These results suggest that among CD8^+^ T_G_ the PD-1^+^ KLRG-1^−^ and PD-1^−^ KLRG-1^+^ populations are functionally distinct and that CD8^+^ T_HSC_ have a higher proliferating potential than CD8^+^ T_G_ even when PD-1 expression is equivalent.

### Active Proliferation Contributes to the Maintenance of T_G_ during the Chronic Phase

**Figure 4:**
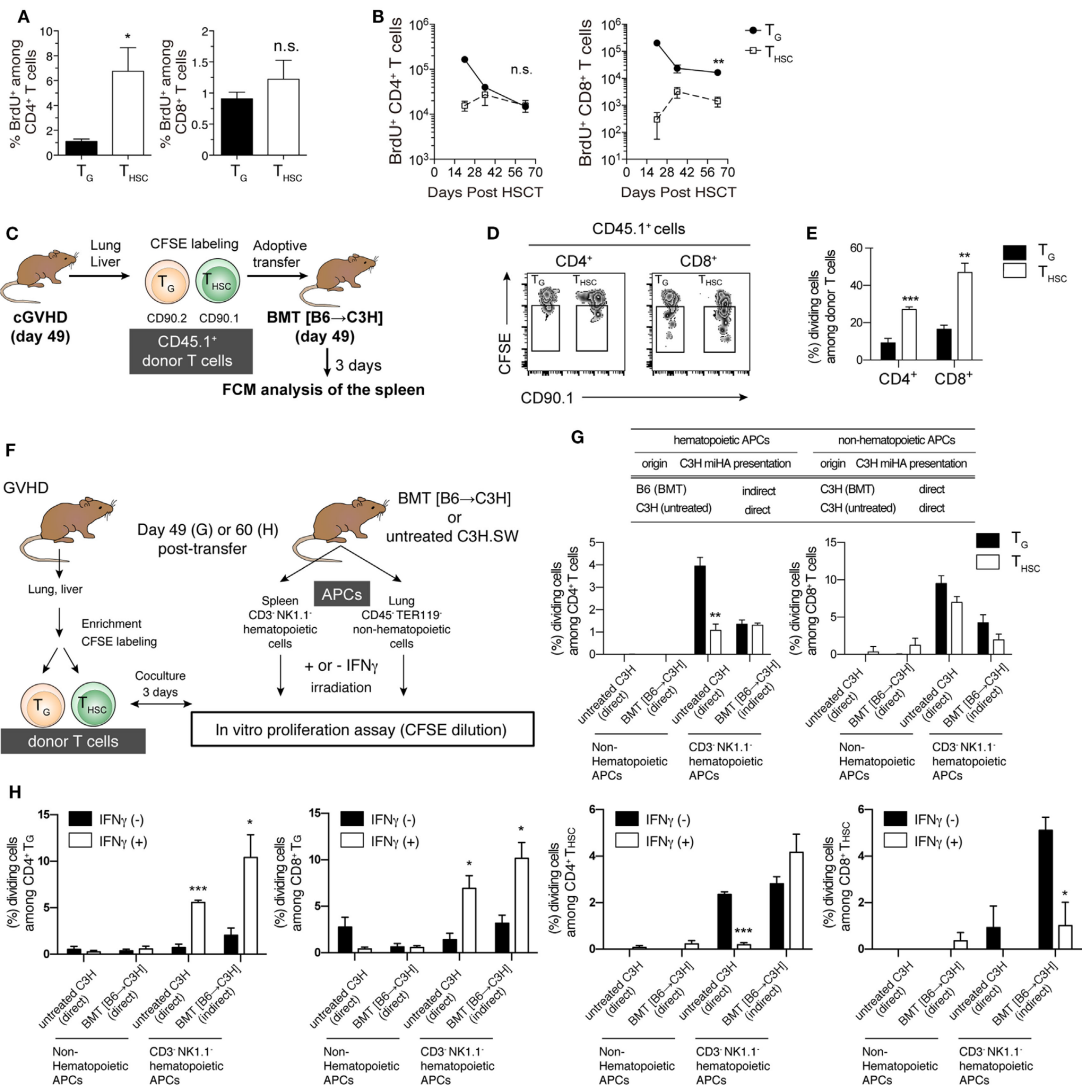
Proliferative potential and allo-recognition pathway of T_G_ and T_HSC_. **(A,B)** Recipients were injected with BrdU 1 h before sacrifice to enable measurement of cell proliferation in liver T cells. **(A)** Percentage of BrdU^+^ CD4^+^ or CD8^+^ T cells within the T_G_ or T_HSC_ populations on day 63. **(B)** Time-course analysis of the BrdU^+^ T_G_ and T_HSC_ populations in the liver. **(C)** Schematic of *in vivo* proliferation assay. CD45.1^+^ Thy1.1^−^ T_G_ or CD45.1^+^ Thy1.1^+^ T_HSC_ were prepared from the liver and lung of chronic graft-versus-host disease (cGVHD) recipients on day 49. These cells were equally mixed, CFSE-labeled, and transferred to phase-matched BMT recipients that had been reconstituted with CD45.2^+^ T-cell-depleted bone marrow (TCD BM). **(D)** Representative flow cytometry plots showing CFSE dilution in CD45.1^+^ donor cells, which consist of Thy1.1^−^ Thy1.2^+^ T_G_ and Thy1.1^+^ Thy1.2^−^ T_HSC_, in the spleen 3 days after secondary transfer. **(E)** Percentages of T_G_ or T_HSC_ that underwent one or more cell division. **(F)** Schematic of *in vitro* proliferation assay. T_G_ and T_HSC_ were cocultured with lung non-hematopoietic cells or spleen CD3^−^ NK1.1^−^ hematopoietic cells, which were prepared from either untreated C3H mice or BMT recipient C3H mice reconstituted with TCD BM from B6 mice, to determine allo-recognition pathway. **(G)** Percentage of T_G_ or T_HSC_ that underwent one or more cell division after 3 days of culture. **(H)** T cells and antigen-presenting cells (APCs) were prepared from cGVHD or BMT recipients on day 60 as described in **(F)**, then APCs were pretreated with 10 U/mL IFNγ for 16 h. Data show the percentage of T_G_ or T_HSC_ that underwent one or more cell division after 3 days of culture. Data represent mean ± SEM [*n* = 6 **(A,B)**, *n* = 4 **(E)**, *n* = 3 **(G,H)**] from one of two independent experiments. *P ≤ 0.05; **P ≤ 0.01; ***P ≤ 0.001 [**(B,E,G)**; T_G_ vs. T_HSC_, **(H)**; and IFNγ (−) vs. IFNγ (+)].

We next investigated the proliferative responses of T_G_ and T_HSC_ by 1 h *in vivo* BrdU labeling on day 63 after allo-HSCT. The proportion of proliferating (BrdU^+^) cells among liver-infiltrating CD4^+^ T cells was significantly lower within the T_G_ population than in the T_HSC_ population, whereas there was no significant difference in the proportion of BrdU^+^ CD8^+^ T cells (Figure 4A). However, in terms of cell number, CD4^+^ BrdU^+^ T_G_ were present in equivalent numbers to CD4^+^ BrdU^+^ T_HSC_, while BrdU^+^ CD8^+^ T_G_ far outnumbered BrdU^+^ CD8^+^ T_HSC_ (Figure 4B). To further investigate the proliferative potential of T_G_ and T_HSC_, we prepared T_G_ and T_HSC_ populations from the liver and lung of cGVHD mice and adoptively transferred them into phase-matched BMT recipients on day 49 after allo-HSCT (Figure 4C). A CFSE-dilution assay demonstrated that a significant proportion of CD4^+^ and CD8^+^ T_G_, and an even greater proportion of T_HSC_ proliferated 3 days after transfer into secondary BMT recipients (Figures 4D,E). These results demonstrate that T_G_ maintain proliferative potential during the chronic phase.

### T_G_ Recognize Host Antigens through both Direct and Indirect Pathways

In the chronic phase of GVHD models, APCs are classified into host radioresistant non-hematopoietic cells or donor BM-derived hematopoietic cells. The former directly present complexes of host-type minor histocompatibility antigen (miHA)-derived peptide and MHC molecule to donor T cells, whereas the latter uptake host-type miHA from host radioresistant non-hematopoietic cells and present miHA-peptide/MHC complexes to donor-T cells through an indirect pathway. To investigate the allo-recognition pathway that contributes to the maintenance of T_G_ and T_HSC_ populations after allo-HSCT, we cocultured T_G_ or T_HSC_ with BMT recipient-derived APCs 49 days after allo-HSCT. To examine the direct pathway, we used radio-resistant host-derived non-hematopoietic cells that present endogenous host-type antigens (Figures 4F,G). To examine the indirect pathway, we used donor-derived T cell/NK cell-depleted hematopoietic cells containing professional APCs, which uptake and then present host antigens. We also used untreated C3H.SW mouse-derived hematopoietic cells and non-hematopoietic cells to examine the direct pathway that is involved in the early induction of effector T_G_. CFSE-dilution assays demonstrated that neither CD4^+^ nor CD8^+^ T_G_ proliferated in response to coculture with non-hematopoietic cells prepared from BMT recipient or untreated C3H.SW mice. In contrast, both CD4^+^ and CD8^+^ T_G_ proliferated when cocultured with hematopoietic cells from C3H.SW mice (direct pathway) and to a lesser extent when cocultured with hematopoietic cells from BMT recipients (indirect pathway) (Figure 4G). T_HSC_ displayed a similar proliferative response except that a small proportion of CD8^+^ T_HSC_ proliferated in response to coculture with non-hematopoietic cells. Considering that host-type hematopoietic APCs are completely absent in mice suffering cGVHD (data not shown), these results suggest that indirect host antigen presentation by donor-derived hematopoietic cells may be involved in the maintenance of T_G_ in cGVHD-affected organs.

Since inflammatory factors such as IFNγ modify antigen processing by inducing expression of immunoproteasomes and MHC molecules (28), we performed similar donor T cell and APC *ex vivo* coculture experiments with APCs that had been pretreated with 10 U/mL IFNγ for 16 h, which induced the expression of MHC class I and MHC class II on both hematopoietic and non-hematopoietic APCs. IFNγ pretreatment did not affect antigen presentation by non-hematopoietic APCs (Figure 4H). However, IFNγ pretreatment of hematopoietic APCs significantly increased the proliferation of CD4^+^ and CD8^+^ T_G_ in both direct and indirect pathway-dependent settings. In contrast, IFNγ pretreatment of hematopoietic APCs significantly suppressed the direct pathway-dependent proliferation of CD4^+^ T_HSC_ and indirect pathway-dependent proliferation of CD8^+^ T_HSC_. These results suggest that IFNγ expression in cGVHD-affected tissues may cause prolonged dominance of T_G_ among CD8^+^ T cells.

### Selective Depletion of T_G_ or T_HSC_ Failed to Ameliorate the Pathology of cGVHD

**Figure 5:**
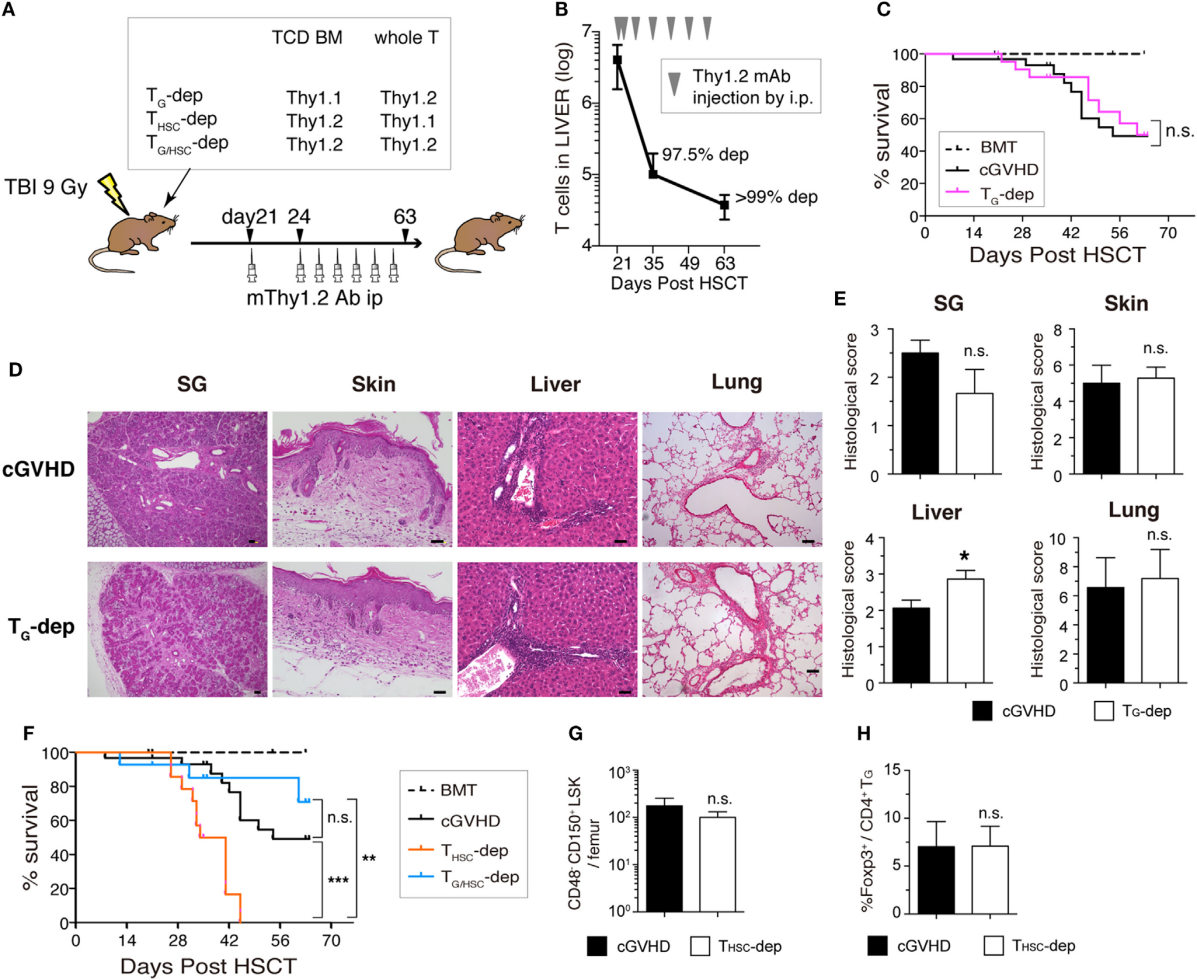
Both T_G_ and T_HSC_ are involved in the pathogenesis of chronic graft-versus-host disease (cGVHD). **(A)** Schematic of the *in vivo* depletion study. T_G_ (T_G_-dep), T_HSC_ (T_HSC_-dep), or both T_G_ and T_HSC_ (T_G_/_HSC_-dep) were selectively depleted from cGVHD mice using the Thy1.1/1.2 congenic markers and an anti-Thy1.2 depleting mAb. Recipient mice received Thy1.1^+^ T-cell-depleted bone marrow (TCD BM) and Thy1.2^+^ splenic T cells, Thy1.2^+^ TCD BM and Thy1.1^+^ splenic T cells, or Thy1.2^+^ TCD BM and Thy1.2^+^ splenic T cells from B6 donors and were then treated with an mThy1.2 antibody or a control Ab on days 21 and 24 after transplantation, and weekly thereafter until day 63. **(B)** Depletion efficiency of anti-Thy1.2 mAb on Thy1.2^+^ T cells in the liver. **(C)** Survival data are combined from three independent experiments (*n* = 12–15). **(D)** Representative HE images and **(E)** histological scoring of the salivary glands, skin, liver, and lung from T_G_-dep and cGVHD (*n* = 5) mice on day 63. Scale bars, 50 µm. **(F)** Survival of cGVHD, T_HSC_-dep, and T_G_/_HSC_-dep group mice. Data are combined from three independent experiments (*n* = 12–15). **(G)** Number of HSCs (CD48^−^ CD150^+^ LSK) in the BM on day 28 after transplantation (7 days after T_HSC_ depletion). **(H)** Proportion of Foxp3^+^ cells within the liver CD4^+^ T_G_ population on day 28 after transplantation. Data in **(B,E,G,H)** represent mean ± SEM (*n* = 3 for the cGVHD group; *n* = 4 for the T_HSC_-dep group) from one of three independent experiments. n.s., not significant; *P ≤ 0.05; **P ≤ 0.01; and ***P ≤ 0.001 (comparisons as indicated).

To determine the involvement of T_G_ and T_HSC_ in the pathogenesis of cGVHD, we selectively depleted T_G_, T_HSC_, or both T_G_ and T_HSC_ from cGVHD mice using Thy1.1/1.2 congenic markers and an anti-Thy1.2 depleting mAb (Figure 5A). This protocol resulted in >95% depletion of Thy1.2^+^ T cells from cGVHD-affected organs from day 35 to the end of our experiments (Figure 5B). Unexpectedly, survival rate, fibrotic changes, and inflammatory cell infiltration in cGVHD-affected organs were not ameliorated by the depletion of T_G_ (Figures 5C–E), despite the predominance of functionally competent T_G_ in the cGVHD-affected organs of untreated cGVHD mice. These results suggest that T_G_ alone are not responsible for the development and/or maintenance of the histopathology of cGVHD. On the other hand, survival was markedly reduced in the T_HSC_-depleted group, in which all mice died within approximately 3 weeks of initial antibody treatment (Figure 5F). This lethal exacerbation of GVHD was not due to the depletion of Thy1^+^ HSCs by the anti-Thy1.2^+^ antibody, because the number of CD48^−^ CD150^+^ lineage^−^ c-Kit^+^ Sca1^+^ long-term HSCs was equivalent between the cGVHD and T_HSC_-depleted groups (Figure 5G). In addition, there was no significant difference in the frequency of Foxp3^+^ liver-infiltrating CD4^+^ T cells between the cGVHD and T_HSC_-depleted groups, suggesting that the lethal exacerbation of GVHD in the T_HSC_-depleted group could not be attributed to the loss of Foxp3^+^ regulatory T cells (Figure 5H). Importantly, simultaneous depletion of T_G_ and T_HSC_ rescued cGVHD mice from the lethal exacerbation of disease induced by the depletion of T_HSC_ alone (Figure 5F). These results suggest that neither T_G_ nor T_HSC_ are solely responsible for cGVHD and that the lethal exacerbation of GVHD induced by T_HSC_ depletion is mediated by T_G_.

### T_HSC_ Suppress Activation of T_G_

**Figure 6:**
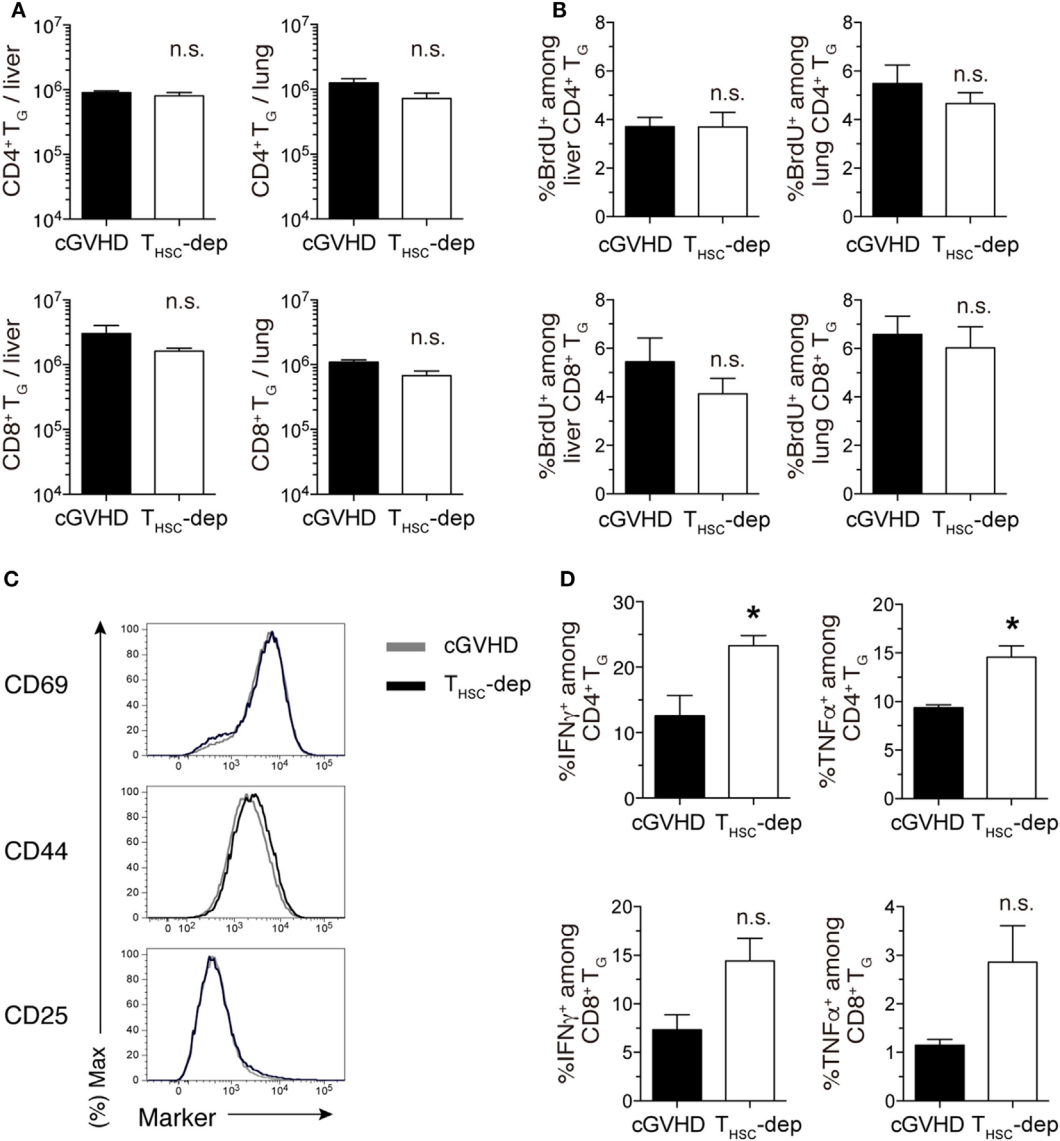
T_HSC_ suppress activation of T_G_. T_HSC_ were selectively depleted from chronic graft-versus-host disease (cGVHD) mice as indicated in Figure 5A. **(A)** Numbers of CD4^+^ and CD8^+^ T_G_ from the liver and lung of T_HSC_-dep and cGVHD on day 28, 7 days after commencing T_HSC_ depletion by mThy1.2 antibody administration. **(B)** Proportion of BrdU^+^ cells within the CD4^+^ or CD8^+^ T cell populations in the liver and lung of T_HSC_-dep and cGVHD mice injected 1 h earlier with 1 mg of BrdU, 28 days after transplantation. Data in **(A,B)** represent mean ± SEM (*n* = 3 for the cGVHD group; *n* = 4 for the T_HSC_-dep group) from one of three independent experiments. **(C)** Representative flow cytometry plots showing the expression of activation markers on CD8^+^ T_G_ from the liver of T_HSC_-dep or cGVHD recipients. **(D)** Liver T cells were collected from cGVHD or T_HSC_-dep group mice and were restimulated with PMA and ionomycin *ex vivo*. Proportions of intracellular IFNγ- or TNFα-positive cells among CD4^+^ and CD8^+^ T_G_. Data represent mean ± SEM (*n* = 3 for the cGVHD group; *n* = 4 for the T_HSC_-dep group) from one of three independent experiments. n.s., not significant; *P ≤ 0.05.

The observation that simultaneous depletion of T_G_ and T_HSC_ ameliorated cGVHD while selective depletion of T_G_ or T_HSC_ failed to ameliorate cGVHD suggests that an interaction exists between these two distinct T cell populations. To investigate this interaction further, we examined the effects of T_HSC_ depletion on the number and function of T_G_. Since T_HSC_-depleted mice died within 3 weeks of depletion (day 21), analyses were performed on days 28 and 35. There were no significant differences in liver and lung T_G_ numbers between the T_HSC_-depleted and cGVHD groups (Figure 6A). In addition, the frequency of BrdU^+^ (proliferating) cells within the T_G_ population and the expression levels of CD44, CD25, and CD69 on T_G_ cells were equivalent between the T_HSC_-depleted and cGVHD groups (Figures 6B,C). However, the proportion of IFNγ^+^ and TNFα^+^ cells among liver-infiltrating CD4^+^ and CD8^+^ T_G_ cells was higher in the T_HSC_-depleted group compared to the cGVHD group on both days 28 and 35 (Figure 6D and data not shown). These results suggest that T_HSC_, despite being 10–100 times lesser in number than T_G_ at the time of depletion, inhibit inflammatory cytokine production by T_G_ and play a critical role in preventing lethal exacerbations of GVHD.

### T_G_ Suppress the Accumulation of T_HSC_ in cGVHD-Affected Organs

**Figure 7:**
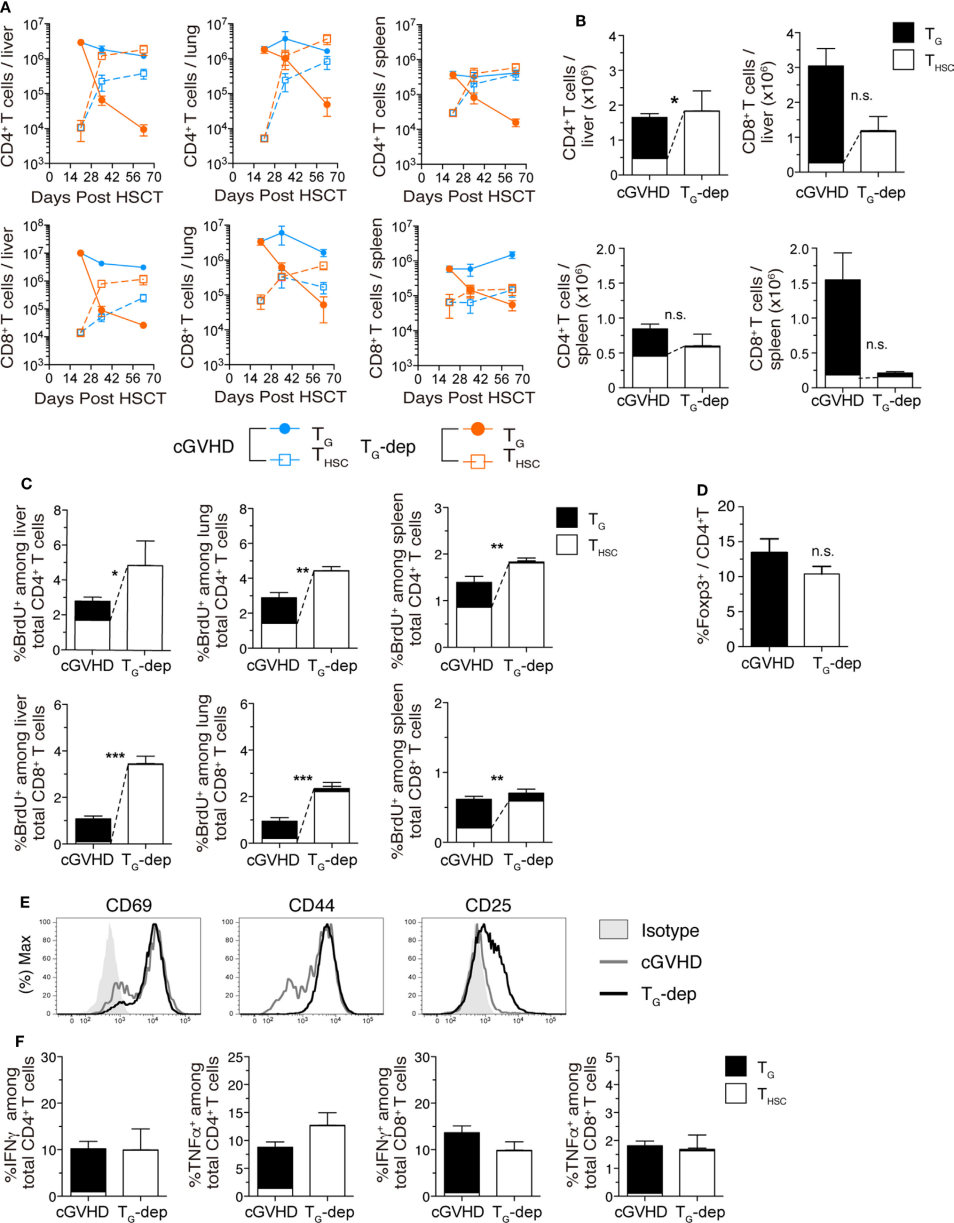
T_G_ suppress accumulation of T_HSC_. T_G_ were selectively depleted from chronic graft-versus-host disease (cGVHD) mice as indicated in Figure 5A. **(A)** Numbers of CD4^+^ and CD8^+^ T_G_ and T_HSC_ in the liver, lung, and spleen of T_G_-dep and cGVHD mice. **(B)** Numbers of CD4^+^ and CD8^+^ T_G_ and T_HSC_ in the liver, lung, and spleen 63 days after transplantation. n.s., not significant; *P ≤ 0.05 (T_HSC_ from cGVHD vs. T_HSC_ from T_G_-dep). **(C)** Proportion of BrdU^+^ cells within the CD4^+^ or CD8^+^ T cell populations in the liver, lung, and spleen of T_G_-dep and cGVHD mice injected 1 h earlier with 1 mg of BrdU, 63 days after transplantation. Data represent mean ± SEM (*n* = 6 for the cGVHD group; *n* = 3 for the T_G_-dep group) from one of three independent experiments. n.s., not significant; *P ≤ 0.05; **P ≤ 0.01; and ***P ≤ 0.001 (T_HSC_ from cGVHD vs. T_HSC_ from T_G_-dep). **(D)** Proportion of Foxp3^+^ cells within the liver CD4^+^ T cell population on day 63 after transplantation. **(E)** Representative flow cytometry plots showing the expression of activation markers on CD8^+^ T_HSC_ from the liver and spleen of T_G_-dep or cGVHD recipients. **(F)** Liver T cells were collected from cGVHD or T_G_-dep group mice and were restimulated with PMA and ionomycin *ex vivo*. Proportions of CD4^+^ and CD8^+^ T_G_ and T_HSC_ positive for intracellular IFNγ and TNFα were analyzed by flow cytometry. Data represent mean ± SEM (*n* = 6 for the cGVHD group; *n* = 3 for the T_G_-dep group) from one of three independent experiments.

Finally, we examined the effects of T_G_ depletion on the number and function of T_HSC_. In kinetic studies of CD4^+^ and CD8^+^ T cell number in the liver, T_G_ rapidly decreased after anti-Thy1.2 mAb treatment in the T_G_-depleted group, whereas T_HSC_ increased to numbers approximately 10-fold higher than the cGVHD group between days 35 and 63 (Figure 7A). By day 63 after allo-HSCT, the number of T_HSC_ (particularly CD4^+^ T_HSC_) in the T_G_-depleted group had increased to levels comparable to the sum of T_G_ and T_HSC_ numbers in the cGVHD group (Figure 7B). To determine whether this increase in T_HSC_ number was due to an increase in T_HSC_ proliferation, we performed BrdU labeling on day 63 (Figure 7C). The proportion of BrdU^+^ cells among liver-infiltrating CD4^+^ and CD8^+^ T_HSC_ was significantly higher in the T_G_-depleted group, outnumbering the sum of BrdU^+^ T_G_ and T_HSC_ in the cGVHD group. There was no significant difference in the frequency of Foxp3^+^ cells among CD4^+^ T cells between the cGVHD and T_G_-depleted groups (Figure 7D). The majority of CD8^+^ T_HSC_ in the liver and lung, but not in the spleen, of T_G_-depleted group mice were CD44^hi^ effector T cells and had increased expression of the activation markers CD25 and CD69 (Figure 7E). The total proportion of IFNγ^+^ or TNFα^+^ cells among liver-infiltrating CD4^+^ and CD8^+^ T cells was equivalent between T_G_-depleted and cGVHD group mice (Figure 7F). However, the cellular composition of these populations switched from predominately T_G_ in the cGVHD group to predominately T_HSC_ in the T_G_-depleted group. Collectively, these results demonstrate that the proliferation and activation of T_HSC_ are augmented by T_G_ depletion in cGVHD-affected organs. This change appears to compensate in part for the decrease in T_G_ numbers and is likely to contribute to the histopathology observed in the cGVHD affected organs of the T_G_-depleted group.

## Discussion

Until now, the contribution of T_G_ to the development and maintenance of cGVHD has remained unclear. In this study, we established a model of cGVHD that recapitulates the pathology of clinical cGVHD. We demonstrated that functional T_G_ persist and predominate over T_HSC_ in the organs affected by cGVHD. Furthermore, our depletion studies revealed the existence of an unexpected bidirectional regulatory interaction between T_G_ and T_HSC_: T_G_ suppress the proliferation and accumulation of T_HSC_ in cGVHD-affected organs, whereas T_HSC_ suppress the activation of T_G_ in affected organs, thereby preventing exacerbations of GVHD mediated by T_G_.

There are few mouse models of cGVHD that accurately recapitulate the histopathology and disease course of clinical cGVHD. Recently, a B6 (H2^b^) Recent (H2^k^) allo-HSCT model, which utilized cyclophosphamide and total body irradiation as a conditioning regimen, was demonstrated to induce fibrotic changes in the lungs, liver, and SG (29, 30). The developmental course of this model recapitulated cGVHD that develops without preceding aGVHD. Mice in our B6→C3H.SW minor mismatched allo-HSCT model also developed several histological aspects of clinical cGVHD, such as fibrotic changes in the skin, liver, lungs, and SG following aGVHD, which may recapitulate the acute to chronic transition in clinical GVHD.

In our cGVHD model, a large number of T_G_ persisted in cGVHD-affected organs up to day 63 after allo-HSCT. On the other hand, peripheral reconstitution of T_HSC_ became obvious from about 4–5 weeks after allo-HSCT and gradually increased thereafter. As a result, both T_G_ and T_HSC_ infiltrated the affected organs in late phase cGVHD with an unexpected predominance of T_G_. In an aGVHD model, Zhang et al. (31) have described a CD44^lo^ CD62L^hi^ IL-7Rα^hi^ CD8^+^ memory stem cell population that maintain CD8^+^ T_G_ in the recipient for a long period of time. In contrast, in our cGVHD model, the majority of T_G_ in affected organs had a CD44^hi^ CD62L^lo^ IL-7Rα^lo^ effector phenotype. Despite the higher proportion of PD-1^+^ or KLRG-1^+^ phenotypically exhausted or senescent populations within the CD8^+^ T_G_ population, the potential for inflammatory cytokine production and *in situ* proliferation was almost equivalent between T_G_ and T_HSC_. Masopust et al. (32) have reported that KLRG-1^+^ PD-1^−^ senescent CD8^+^ T cells induced by a heterologous prime-boost vaccination show strong cytotoxic activity. Thus, KLRG-1^+^ PD-1^−^ CD8^+^ T_G_ may be involved in the cellular immunopathogenesis of cGVHD.

In the cGVHD affected liver and lung, a significant proportion of CD8^+^ T_G_ were actively proliferating even at day 63, suggesting that T_G_ maintenance is a dynamic process controlled by cell proliferation and cell death. Considering that T_G_ do not proliferate in response to direct presentation of host antigens by non-hematopoietic cells, it appears that donor HSC-derived APCs capture host alloantigens and indirectly present them to T_G_ in cGVHD-affected organs. An intriguing question that remains is whether T_G_ clones that expanded in response to direct presentation in the acute phase cross-react to indirect presentation in the chronic phase, or whether indirect presentation-reactive T_G_ clones are independently expanded by donor-derived APCs after repopulation of these cells in the peripheral tissues. A better understanding of the clonal response of T_G_ after allo-HSCT and the pathological significance of this process may facilitate the early diagnosis and treatment of cGVHD.

The finding that depletion of the small T_HSC_ population potentiated the inflammatory cytokine production of T_G_ and caused a T_G_-mediated acute exacerbation of GVHD suggests that reconstitution of T_HSC_ is important for limiting excessive activation of T_G_ and promoting acute to chronic disease transition following allo-HSCT. Impaired inhibition of T_G_ by T_HSC_ may account for the GVHD that is refractory to immunosuppressive therapy with a calcineurin inhibitor, which inhibits both T_G_ activation and T_HSC_ development (33). However, the molecular and cellular mechanisms underlying the inhibition of T_G_ activation by T_HSC_ remain to be elucidated.

Reconstitution of T_HSC_ is markedly delayed and suppressed in the presence of GVHD (16). In the early phase of aGVHD, CD4^+^ T_G_ impair the production of common lymphoid progenitors through the destruction of the hematopoietic niche in the BM, resulting in a severe reduction in T- and B-lymphocyte genesis (17, 34). T_G_ also impair thymopoiesis by disrupting the thymic epithelium (35). Thus, T_G_ may suppress T_HSC_ reconstitution by impairing primary lymphoid tissues. In the present study, depletion of T_G_ resulted in a rapid increase in the number of T_HSC_ in the liver and lung. Interestingly, increased numbers of T_HSC_ in the T_G_-depleted group partially compensated for the decreased numbers of T_G_ in cGVHD-affected organs. This finding points to the possible existence of a niche that provides antigenic signals and survival factors to pathogenic T cells of T_G_ or T_HSC_ origin. Such a “pathogenic T cell niche” might have a fixed pool capacity, meaning that T_G_ and T_HSC_ compete with each other for space in the niche during cGVHD.

In summary, we have characterized the cellular mechanisms underlying the maintenance of pathogenic T cells in a clinically relevant cGVHD model. Both T_G_ and T_HSC_ with the potential to proliferate and produce inflammatory cytokines infiltrated cGVHD-affected organs, with the number and/or activity of T_G_ and T_HSC_ being reciprocally regulated by each other. In addition, our depletion studies highlight the importance of reciprocal tuning of the balance between T_G_ and T_HSC_, which requires the successful reconstitution of T_HSC_, in the control of GVHD. By elucidating the interactions between T_G_ and T_HSC_, our findings will help guide the development of novel therapeutic strategies for the prevention and treatment of cGVHD.

## Ethics Statement

All animal experiments were conducted in accordance with institutional guidelines with the approval of the Animal Care and Use Committee of the University of Tokyo.

## Author Contributions

MK-K, SU, and KM participated in research design; MK-K, SU, JA, SS, FHWS, TM, NS, AY, FS, and WY conducted experiments; MK-K and SU performed data analysis; MK-K, SU, JA, SS, FHWS, MK, YS, MI, TT, and KM wrote or contributed to the writing of the paper.

## Conflict of Interest Statement

The authors declare that the research was conducted in the absence of any commercial or financial relationships that could be construed as a potential conflict of interest.
